# On the complementarity of DNA barcoding and morphology to distinguish benign endemic insects from possible pests: the case of *Dirioxa pornia* and the tribe Acanthonevrini (Diptera: Tephritidae: Phytalmiinae) in Australia

**DOI:** 10.1111/1744-7917.12769

**Published:** 2020-05-08

**Authors:** Francesco Martoni, Isabel Valenzuela, Mark J. Blacket

**Affiliations:** ^1^ Agriculture Victoria Research AgriBio Centre for AgriBioscience Bundoora Victoria Australia

**Keywords:** biodiversity, biosecurity, COI, fruit flies, Agriculture

## Abstract

Fruit flies are considered economically important insects due to some species being agricultural pests. However, morphological identification of fruit fly adults and larvae can be difficult requiring a high level of taxonomic expertise, with misidentifications causing problematic false‐positive/negative results. While destructive molecular techniques can assist with the identification process, these often cannot be applied where it is mandatory to retain a voucher reference specimen. In this work, we non‐destructively (and partial‐destructively) processed larvae and adults mostly belonging to the species *Dirioxa pornia* (Walker, 1849), of the poorly studied nonpest fruit fly tribe Acanthonevrini (Tephritidae) from Australia, to enable molecular identifications whilst retaining morphological vouchers. By retaining the morphological features of specimens, we confirmed useful characters for genus/species‐level identification, contributing to improved accuracy for future diagnostics using both molecular and morphological approaches. We provide DNA barcode information for three species of Acanthonevrini known from Australia, which prior to our study was only available for a single species, *D. pornia*. Our specimen examinations provide new distribution records for three nonpest species: *Acanthonevroides variegatus* Permkam and Hancock, 1995 in South Australia, *Acanthonevroides basalis* (Walker, 1853) and *D. pornia* in Victoria, Australia; as well as new host plant records for *D. pornia*, from kangaroo apple, apricot and loquat.

## Introduction

Correct species‐level identification is key to many fields of entomology, especially pest management, diagnostics, and biosecurity. Flies (Insecta: Diptera), for example, are one of the most important insect groups for economic reasons (White & Elson‐Harris, 1992) and they show very high diversity, with many species currently not well characterized especially at the larval stage (Permkam & Hancock, 1995). In fact, in agriculture and biosecurity, fly larvae are usually initially identified to family level, generally providing enough information to assess economic concerns (Ferrar, 1987; CSIRO, 1991). Within Diptera, Tephritidae is a very large family with nearly 5000 currently recognized species, and the larvae of numerous species are economically important plant pests (White & Elson‐Harris, 1992). Indeed, tephritid larvae cause greater losses to fruit and vegetable production than any other family of Diptera (Ferrar, 1987). However, less than ten percent of Tephritidae species are considered plant pests (Plant Health Australia, 2018) and specimens identified only to a family‐level can lead to false‐positive/false‐negative records of pest species. Species‐level morphological identification of tephritids can be time‐consuming, with processing requiring taxonomic expertise due to the sparse morphological characters available for different life stages, including adults and larvae (White & Elson‐Harris, 1992).

Thirty‐nine Australian species of the tribe Acanthonevrini (Tephritidae: Phytalmiinae) represent a subgroup of fruit flies where all species are considered nonpests (Permkam & Hancock, 1995; Hancock *et al*., 2000). Morphological keys for adult Australian Acanthonevrini are available (Permkam & Hancock, 1995; Hancock, 1996), but appear to have not been widely used to date (see below), and identification of immature flies remains even more challenging due to a lack of taxonomically informative morphological characters of diagnostic importance available, and with larvae from only the most commonly collected species, the Island fly *Dirioxa pornia*, having been characterized to date (White & Elson‐Harris, 1992).

Molecular identification through DNA barcoding (Hebert *et al*., 2003) is a valuable method to complement morphology to accurately identify fly larvae to species (e.g., Blacket *et al*., 2012, 2015; Martoni *et al*., 2019) and can be easily employed partially destructively by using single appendages from adult insects (i.e., a leg), retaining the rest of the body. When DNA barcoding is paired with non‐destructive DNA extraction methods it allows, at least partially, preservation of the morphological features of the specimen (e.g., Castalanelli *et al*., 2010; Martoni *et al*., 2019). However, an accurate identification is strongly dependent on the presence of DNA sequences on public online databases, such as GenBank (Benson *et al*., 2018) and BOLD (Ratnasingham & Hebert, 2007). The presence of sequences linked to accurately identified specimens is fundamental (Hodgetts *et al*., 2016), as missing data, or data with incorrect annotations, can be extremely detrimental (e.g., Bengtsson‐Palme *et al*., 2016; Mioduchowska *et al*., 2018). Hence, both a valid morphological identification and the production of viable DNA sequences is widely considered of high importance, especially in those instances where either one or the other result were challenging to acquire (e.g., Ghorbani *et al*., 2017; Kanturski *et al*., 2018; Martoni *et al*., 2018). In the current study, DNA barcoding analysis was performed on Acanthonevrini adult and larval samples preserved in entomological collections, to enable taxonomic identification to species level and to link larval specimens to adults.

The aims of the current study were: (i) to identify specimens of *D. pornia* available in entomological collections and separate them from other Acanthonevrini specimens and fruit flies using cytochrome c oxidase I (COI) DNA barcoding (when reference sequences were available on public databases), (ii) to not compromise morphological features and retain physical voucher specimens, (iii) to confirm taxonomically informative morphological characters of adults and larvae, (iv) to use the DNA sequences obtained to assess Australian *D. pornia* haplotype diversity, and (v) to generate additional COI DNA barcode sequences to increase the number of Acanthonevrini species currently available in online reference databases.

## Materials and methods

A total of 72 Tephritidae, representing 15 adults and 57 larvae, were examined for this study. All larvae were from the Victorian Agricultural Insect Collection (VAIC, https://collections.ala.org.au). Adult specimens from entomological collections included six ethanol‐preserved specimens from the South Australian Museum (SAM), three specimens from the Queensland Primary Industries Insect Collection (QDPC), and six specimens from the VAIC (Table S1). A subset of these adults, one specimen of *Acanthonevroides basalis* (VAIC) and one specimen of *Termitorioxa termitoxena* (Bezzi, 1919) (QDPC), was examined further for molecular analyses (Table S1).

Adult specimens were initially identified morphologically using Permkam & Hancock, 1995. The fly larvae, of various instars, were initially identified morphologically (using White & Elson‐Harris, 1992) as being Island flies, *D. pornia* (Table S1) based on the known characteristics of third instar larvae, including the morphology of the anterior and posterior spiracles, oral ridges, and anal lobes (White & Elson‐Harris, 1992). These specimens were collected through routine fruit fly surveillance activities (Dominiak & Daniels, 2012) in the state of Victoria from locally grown produce and from interstate (New South Wales and Queensland) intercepted produce (Table S1). All larval specimens were preserved after collection into absolute ethanol and then stored at –20 °C at the Victorian Agricultural Insect Tissue Collection (VAITC), located at Agribio, Bundoora, Australia, within the VAIC. We asked whether amongst the tentatively identified *D. pornia* larvae there were *Acanthonevroides* and *Termitorioxa* as it had occurred with the adults (see below). This was a possibility given that larvae of other Acanthonevrini genera have not been described and many of our studied larvae were much smaller than third instars.

High resolution automontage images of a larva in ethanol post DNA extraction and adult flies were obtained using the Leica Application Suite software (version 4.5.0), from 20 to 30 stacked images obtained using a Leica stereo microscope M205C with a DFC450 camera.

For DNA extractions from adult flies, one leg was detached from each specimen and placed in a 1.5 mL Eppendorf microtube and used for destructive DNA extraction using the DNeasy Blood and Tissue Kit (Qiagen, Hilden, Germany). Final elution of the DNA extraction was 100–150 μL. For larvae, DNA extractions were performed using the non‐destructive method presented in Martoni *et al*. (2019). After DNA extraction, each larval specimen was transferred into 70% ethanol and re‐deposited in the VAIC as morphological voucher specimens (Plant Health Australia, 2018). DNA was stored at −20 °C.

Polymerase chain reaction (PCR) was performed on larval specimens using the same protocol, kit and cycle presented in Martoni *et al*. (2019), together with the universal COI primers, LCO1490 (5′‐GGTCAACAAATCATAAAGATATTGG‐3′) and HCO2198 (5′‐TAAACTTCAGGGTGACCAAAAAATCA‐3′) (Folmer *et al*., 1994). For adult flies, generally older specimens with more degraded DNA, a shorter fragment of approximately 350 bp of COI was targeted using the new reverse primer FFCOI‐R (designed here based on sequences available online; TSCCAGCYCCRTTTTCHAC), used in combination with LCO1490. PCR amplification was confirmed on 2% agarose gels. All amplified PCR products were purified, and Sanger sequenced both directions commercially by Macrogen Inc. (Seoul, Korea), on an ABI sequencer.

The sequences generated for this study were aligned with all publicly available sequences of *D. pornia* from Western Australia (*N* = 11, DQ116377‐79; KT864810‐17), South Australia (*N* = 5, KT864800‐01; KT864807‐09), and Queensland (*N* = 5, KT864802‐06) from GenBank and BOLD. Alignment of sequences was performed using the software MEGA X (Kumar *et al*., 2018) and the ClustalW algorithm. The barcode of life database (BOLD; Ratnasingham & Hebert, 2007) was also used to query each sequence to provide molecular identification of specimens. All sequences obtained here have been submitted to GenBank (National Centre for Biotechnology Information, NCBI), accession numbers MK265278‐MK265326 and MK955795‐MK955800 (Table S1). PopArt (Leigh & Bryan, 2015) was used to construct a Median Joining haplotype network (ε = 0) (Bandelt *et al*., 1999) using all the sequences.

## Results

**Fig. 1:**
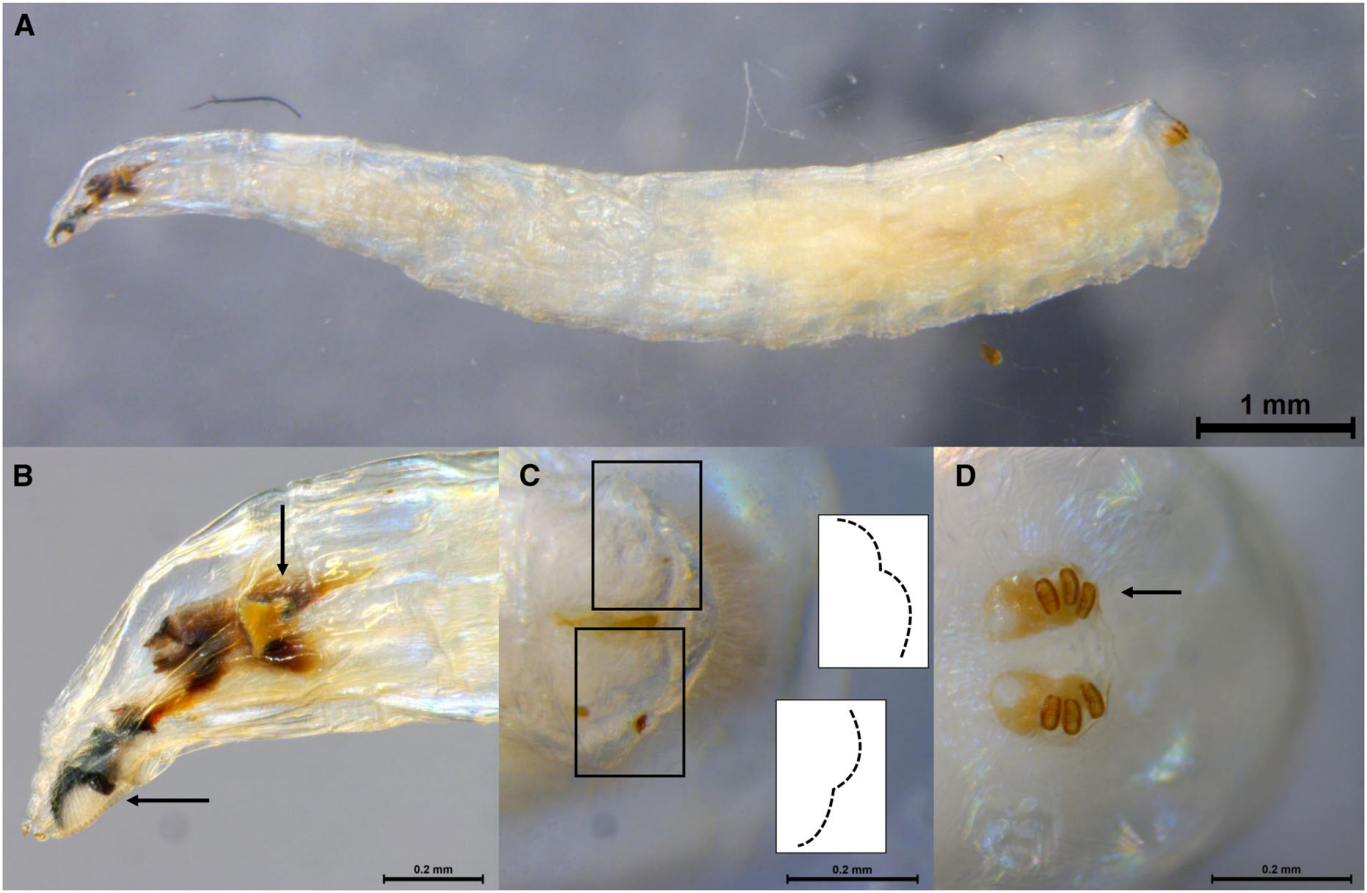
*Dirioxa pornia*, larva (VAITC4606), after non‐destructive DNA extraction. (A) Habitus (lateral). (B) Head (lateral), posterior spiracles and oral ridges indicated by arrows. (C) Anal‐plate (ventral), bilobes indicated in boxes. (D) Posterior spiracles (dorsal), indicated by arrow. Scale bars measure 1 mm (A) and 0.2 mm (B–D).

All larval specimens analyzed retained their general morphological features (Fig. 1), presenting a general discoloration only in the areas of the head and posterior (Fig. 1A, B), as previously demonstrated when using this method with Muscidae (Martoni *et al*., 2019). Hence, the specimens could be re‐deposited as morphological vouchers in the VAIC after DNA sequences were obtained. Of the total 57 larval samples 49 were successfully sequenced and generated 621 bp long sequences (MK265278–MK265326). All of these were confirmed to be *D. pornia* with a COI sequence similarity between 99% and 100% to each other and to sequences available on GenBank and BOLD (Fig. S1), therefore considered a strong species match (Ratnasingham & Hebert, 2007).

**Fig. 2:**
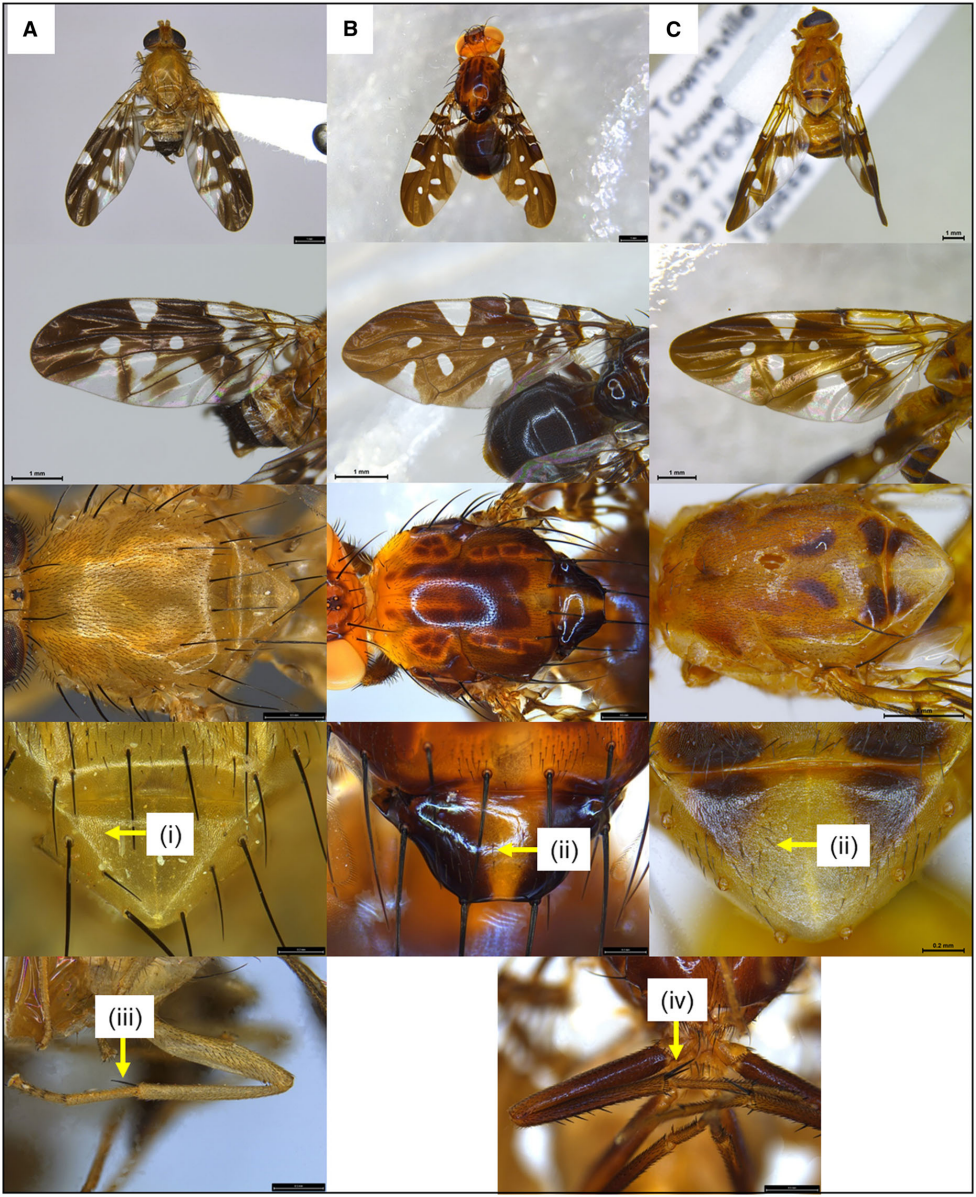
Morphology of the genera *Dirioxa, Acanthonevroides* and *Termitorioxa*. (A) *Dirioxa pornia* (VAIC) showing (i) lack of setae on scutellum and (iii) single apical spine on mid‐tibia; (B) *Acanthonevroides variegatus*, (SAM), and (C) *Termitorioxa termitoxena*, (QDAF), with both genera showing: (ii) a setose scutellum and (iv) two strong mid‐tibial apical spines (iv = image shows *A. variegatus*).

When considering the morphology of adult specimens (Permkam & Hancock, 1995), besides *D. pornia*, two other commonly collected Acanthonevrini genera, *Acanthonevroides* (which has 5 described species) and *Termitorioxa* (which has 7 described species), were both often misidentified as Island flies in state museums and other reference collections (Blacket unpublished data). Based on this result, the key morphological characters that separate the genera *Dirioxa* from *Acanthonevroides* and *Termitorioxa* (Permkam & Hancock, 1995; Hancock, 1996) have been highlighted in Figure 2. Furthermore, in order to further characterize these three genera, six adult insects were selected as representative and DNA was extracted from single legs. Viable COI sequences were generated (Acc. Numbers MK955795–MK955800, Table S1) confirming the identification of *D. pornia* (*N* = 4) based on strong matches to sequences available on GenBank and BOLD, as well as the first partial COI sequences for *Acanthonevroides basalis* (*N* = 603 bp) and *Termitorioxa termitoxena* (*N* = 324 bp) (not previously present on GenBank or BOLD). These have been uploaded on GenBank after comparing them to all the other Phytalmiinae sequences to confirm that the sequences indeed belong to the subfamily (Fig. S1). The large genetic differences between COI sequences of these species obtained from voucher specimens of *A. basalis* and *T. termitoxena*, together with the retained specimen vouchers both for adults and for larvae of *Dirioxia*, helped confirm the validity of the morphological characters distinguishing *Dirioxa* from the specimens belonging to the genera *Acanthonevroides* and *Termitorioxa* (Fig. 2).

**Fig. 3:**
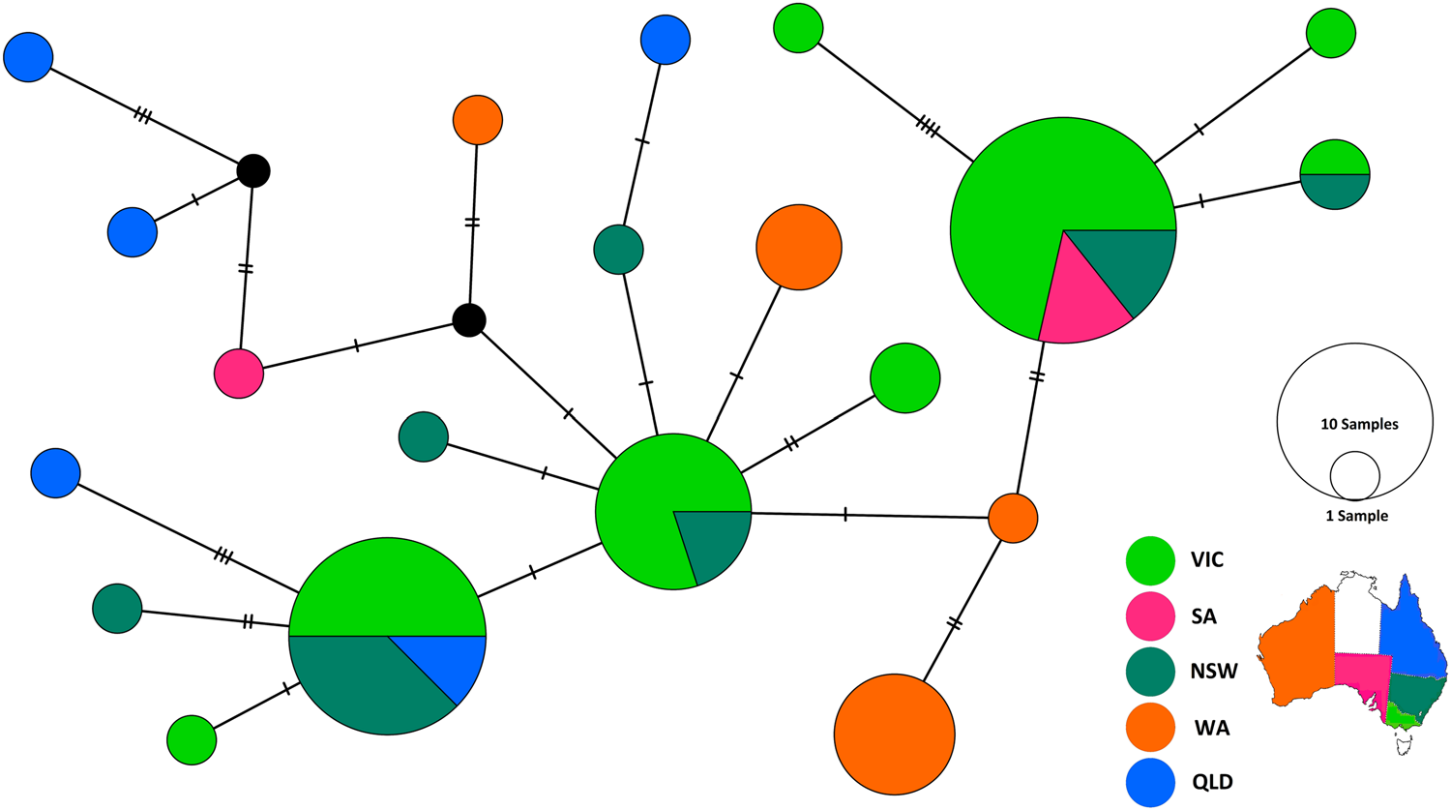
Median joining network including 73 sequences of *Dirioxa pornia*. Haplotypes are color‐coded according to the geographical origin of the Australian populations. A circle represents a haplotype, which varies in size depending on the number of samples, while black circles represent intermediate haplotypes that have not been sampled or are missing.

The new molecular data from *D. pornia* generated in this project (49 sequences) was assessed in a haplotype network (Fig. 3) together with sequences already present on GenBank and BOLD (24 sequences). This revealed a total of 20 different haplotypes. Two were present in South Australia (Fig. 3, pink), four in Western Australia (Fig. 3, orange), five in Queensland (Fig. 3, blue), seven in New South Wales (Fig. 3, dark green), and eight in Victoria (Fig. 3, light green). Of these, only four haplotypes were shared between different States. All the shared haplotypes were present both in Victoria and in New South Wales, while two haplotypes were also shared with Queensland and South Australia, respectively (Fig. 3).

In terms of hosts, the collections of *D. pornia* showed a wide host range, as previously recorded in the literature, with *Citrus* spp. being the most common host followed by pome fruits and avocado (Table S1). In addition, three new hosts were detected in this study: one Solanaceae, Kangaroo apple, *Solanum aviculare* G. Forster; and two Rosaceae: apricot, *Prunus armeniaca* L., and loquat, *Eriobotrya japonica* (Thunb.) Lindl. All these records were from Victoria (Table S1).

## Discussion

### Morphology and COI barcoding enable better species identifications

Adult flies of several Acanthonevrini species that are very similar to *D. pornia* have been misidentified as Island fly in most reference collections examined. The two most common genera being *Acanthonevroides* and *Termitorioxa*, which after *D. pornia* are the most common Acanthovevrini collected in southern/eastern and northern Australia, respectively. These misidentifications are likely due to the very similar wing patterns in these species (Permkam & Hancock, 1995) (Fig. 2).

The identification of species within this relatively poorly studied group not only presented challenges due to the limited diagnostic distinguishing characters for each species, but it was also made even harder by a profound lack of DNA sequences in any database. In fact, while sequences could be found for *D. pornia*, no sequences were available for any of the other Acanthonevrini species from Australia. Our study provides complete and partial DNA barcodes for two additional species, *A. basalis* and *T. termitoxena*, respectively, for which there were no reference sequences previously. The importance of these new sequences is reflected by the fact that prior to our study the closest matches on BOLD (<89% match) for these two species would have been with exotic tephritid species of the genera *Anastrepha* and *Ceratitis*, respectively (Table S1), highlighting the urgent need for more reference sequences from Australia's lesser studied endemic nonpest fruit flies. Therefore, the results presented here add value to both sides of the complementary morphology/barcoding species identification process.

DNA barcoding successfully confirmed the initial morphological diagnostic identification of both large and small fly larvae collected from overripe/rotten fruit as *D. pornia*, confirming once more how this technique can complement morphology‐based taxonomy. On the other hand, the initial morphological examination of adult flies which led to the misidentification of multiple Acanthonevrini species contributed to generating DNA sequences never obtained before, improving online databases that are now available for future works. Hence, this work confirmed the complementarity between COI barcoding and morphology for insect species identification.

Indeed, DNA barcoding was successfully adopted for the identification of larvae and adults of *D. pornia* and their separation from specimens of other genera. Species‐level identification for *D. pornia*, with strong matches with sequences available on BOLD, allowed us to extend knowledge of its distribution to the state of Victoria, where it was not previously formally reported in the scientific literature (e.g., Hancock *et al*., 2000; Hancock, 2013; Plant Health Australia, 2018). Despite Island fly specimens having been collected from Victoria for decades (Plant Health Australia, 2001), a formal taxonomic/genetic identification of this species in Victoria had not been conducted prior to our study, leading to it not being reported in the most recent fruit fly checklist (Plant Health Australia, 2018). The new molecular data presented here revealed a lack of geographic structure for *D. pornia* across Australia, with much of the species genetic variation reported here being present across multiple states. Whilst most diversity appeared to be present in New South Wales and Victoria (>50% of haplotypes), this is probably a result of the limited sampling from other locations in Australia. For example, the specimens from the historically introduced population from Western Australia do not share haplotypes with any other Australian state, suggesting that the introduction might have been an isolated event, from a population that is so far unsampled.

**Fig. 4:**
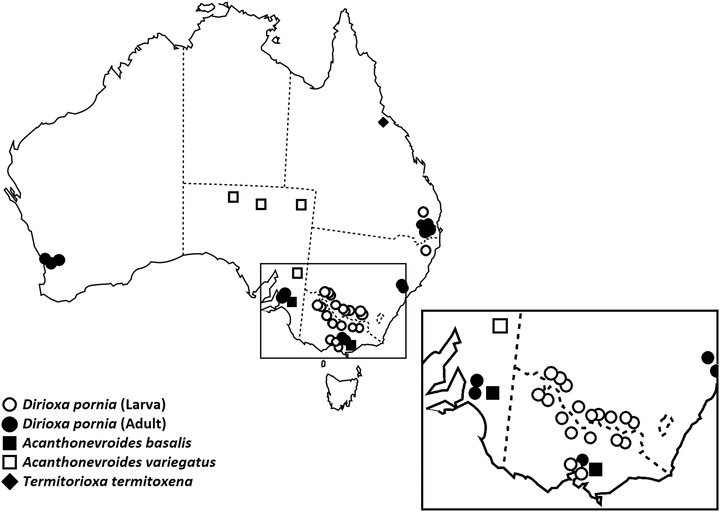
Distribution of the fly adults and larvae included in this study (examined for molecular and/or morphological variation). Each dot represents the location where one or more specimens were collected. For number of specimens from each location see Table S1.

Contemporaneously, being able to associate the molecular data generated here with the preserved morphological voucher specimens, enabled better‐informed morphological examinations, as incorrect identifications could provide false‐positive records for biosecurity and border controls when benign endemic species are mistakenly identified as pest species (Plant Health Australia, 2018). At least four different Acanthonevrini species are known to have been collected by fruit fly Cue lure traps (Hancock, 1996; Plant Health Australia, 2018), with some of these specimens previously misidentified as Island flies in collections. To improve adult fruit fly identification the key morphological characters that differentiate *Dirioxa* (Island fly) from the genera *Acanthonevroides* and *Termitorioxa*, as reported by Permkam and Hancock (1995) and Hancock (1996), are highlighted here through images for the first time (Fig. 2), with *Dirioxa* possessing a single long mid‐tibial apical spine, reduced setae on the scutellum, as well as coloration and wing pattern differences (Fig. 2). Our morphological examination of reference collection specimens has provided additional geographic distribution records for two other Acanthonevrini species. *Acanthonevroides basalis* was previously known only from South Australia, we report it here to be present from at least one location in Victoria (Fig. 4, Table S1). While *A. variegatus*, previously known from Western Australia, the Northern Territory and Queensland is reported for the first time from South Australia (Fig. 4, Table S1).

The two new DNA sequences obtained for *Acanthonevroides* and *Termitorioxa*, being the first samples for their respective genera, also provide reference sequences for future studies to enable molecular identification of these groups. This substantially improves future adoption of the DNA barcoding and metabarcoding approaches to species identification in biosecurity and plant protection. For example, the potential use of metabarcoding analyses in pest detection programs requires the availability of COI DNA sequences on public databases in order to have a correct taxonomical identification (Piper *et al*., 2019). Furthermore, the DNA extraction method used here could be easily implemented in a high throughput metabarcoding workflow since it allows processing of multiple samples at the same time and provides enhancement of morphological features without requiring dissections for each single specimen (Martoni *et al*., 2019; Piper *et al*., 2019).

### The misidentification of the Acanthonevrini and the risks related to plant protection

Surveillance programs for fruit fly adults and larvae in fruit fly pest‐free areas have been conducted long‐term in Australia to provide ongoing, accurate distribution records and assist with pest management (Dominiak & Daniels, 2012). The specimens examined in our study now provide local reference DNA sequences and morphological voucher specimens to assist with future surveys.

The larval stages of only one species of Acanthonevrini have been characterized morphologically (White & Elson‐Harris, 1992) making the morphological identification of possible pests difficult. The only Acanthonevrini species so far morphologically characterized, the nonpest Island fly, utilizes a wide range of host fruit, raising the risk of them being misidentified as other pest tephritid species (Plant Health Australia, 2018). All other Acanthonevrini species appear to either utilize a single type of fruit (i.e., fig fruit) or breed under the bark of trees and fallen logs (Permkam & Hancock, 1995), except for an earlier association of *Termitorioxa* with termite galleries, which was later considered to be only incidental (Hancock, 2002).

Unfortunately, this poor characterization of larval morphology and the lack of a precise description of the larvae of other species of Acanthonevrini, makes larval morphological comparisons and identifications complex, if not impossible. Here we confirmed that the characters currently in use for morphological identification of *D. pornia*—the anterior spiracles, the oral ridges and the anal‐plate morphology (White & Elson‐Harris, 1992)—are preserved post nondestructive DNA extraction (Fig. 1). Additionally, the DNA extraction method adopted here cleared the specimens in the area of the head, allowing the morphological examination of internal structures such as the mouth hooks (Fig. 2B), without the need of dissections, as previously demonstrated for muscid flies (Martoni *et al*., 2019). However, due to the lack of descriptions for other species’ larvae, the use of COI DNA barcoding is the only method allowing identification of other species, especially in a high throughput context.

Here, DNA barcoding extended the known distribution of the most commonly collected Acanthonevrini species, *D. pornia*, to include Victoria, the most southerly state in mainland Australia (Fig. 4, Table S1). This examination also confirmed additional information on the host plant use, with three new host records—kangaroo apple, *Solanum aviculare* (Forster, 1786), apricot *Prunus armeniaca*L., and loquat, *Eriobotrya japonica* (Thunb.) Lindl. (Table S1)—documented in this study. Kangaroo apple is a widespread native solanaceous plant most abundant along the east coast of Australia (ALA, 2019). This host being a noncrop perennial plant could act as a natural reservoir for this species. Apricot and loquat are residentially and commercially grown in Australia. Even though *D. pornia* is not considered a pest, with previous data suggesting that these flies breed on overripe/fallen fruits only (Plant Health Australia, 2018), it is important that growers of these crops know that this tephritid species could be found in their orchards.

The risks associated with the misidentification of Acanthonevrini species is due to: (i) their strong larval morphological similarity to serious tephritid fruit fly pest species (e.g., *Bactrocera* sp.), (ii) to the fact that *D. pornia* can be found in overripe commercial fruits, and (iii) that adults of *D. pornia* and other genera examined here can end up in insect traps in the field, potentially generating false‐positive results in pest control management strategies. Nonetheless, *D. pornia* is a nonpest polyphagous fly, known to feed on a variety of plants but only on overripe, damaged or fallen fruits (Plant Health Australia, 2018). With the addition of apricots and loquat (both Rosaceae), and kangaroo apple (Solanaceae), this species has now been recorded on 86 hosts in 28 families (Plant Health Australia, 2018). This species is not known to have established in Tasmania, but has been introduced to Perth, Western Australia (Plant Health Australia, 2018). Its updated distribution (current study) now includes the whole of mainland eastern Australia, from the Cape York Peninsula (Queensland) to Melbourne (Victoria).

## Disclosure

The authors declare that they have no conflicts of interest.

## Supporting information


**Fig. S1**. Neighbour Joining tree based on ∼620 bp of COI of *D. pornia* from our study and the newly sequenced COI of *T. termitoxena* (324 bp) and *A. basalis* (603bp) compared to all currently available species of Phytalmiinae from BOLD and GenBank. Scale bar represents 2% sequence difference.

**Table S1**. Samples used for this study. Information includes number of samples, VAITC number, date of collection, state and locality, host plant, provenance, morphological and DNA barcode assessment, specimen type, morphological and molecular identification, and GenBank accession numbers.
