# Supplementary figures and images for: On the complementarity of DNA barcoding and morphology to distinguish benign endemic insects from possible pests: the case of Dirioxa pornia and the tribe Acanthonevrini (Diptera: Tephritidae: Phytalmiinae) in Australia
Source: Insect Sci. 2020 May 8;28(1):261–70. doi: 10.1111/1744-7917.12769 (PMC7818419; doi:10.1111/1744-7917.12769)

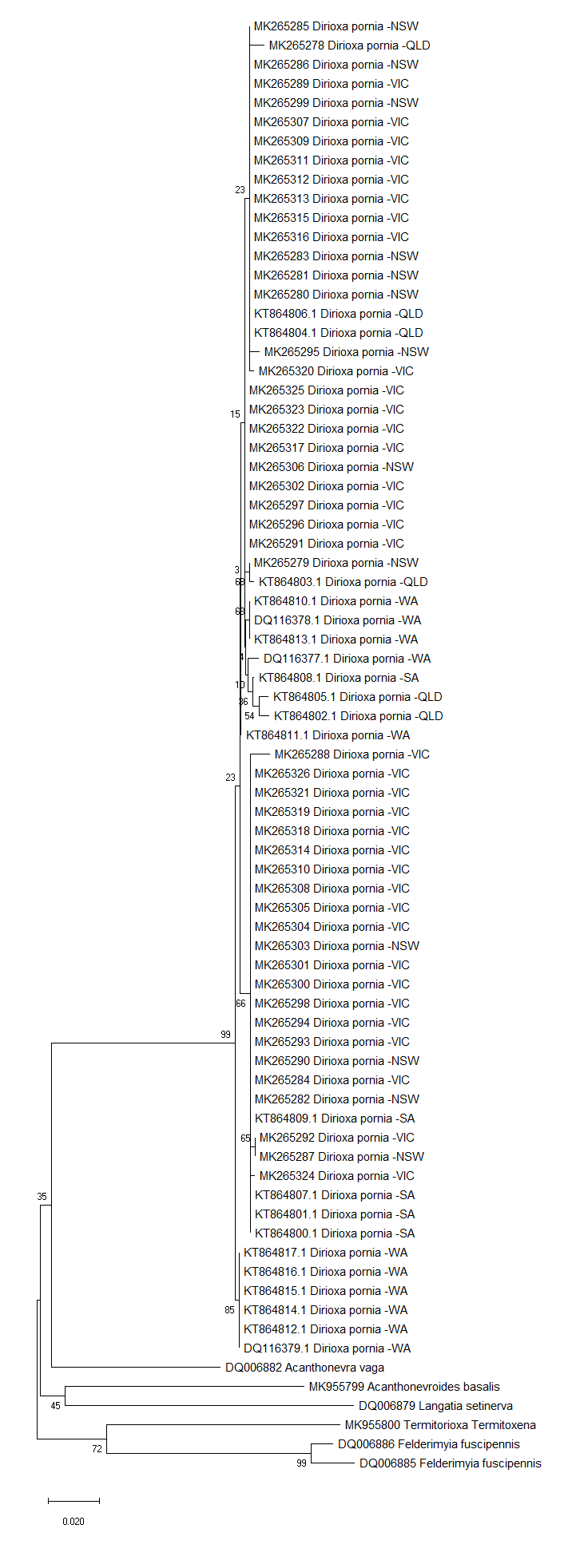

Supplement: Supplementary file 1 — Fig. S1. Neighbour Joining tree based on ∼620 bp of COI of D. pornia from our study and the newly sequenced COI of T. termitoxena (324 bp) and A. basalis (603bp) compared to all currently available species of Phytalmiinae from BOLD and GenBank. Scale bar represents 2% sequence difference. [file INS-28-261-s001.tif]
